# The molecular characterization of fixed inversions breakpoints unveils the ancestral character of the *Drosophila guanche* chromosomal arrangements

**DOI:** 10.1038/s41598-018-37121-5

**Published:** 2019-02-08

**Authors:** Dorcas J. Orengo, Eva Puerma, Montserrat Aguadé

**Affiliations:** Departament de Genètica, Microbiologia i Estadística, Facultat de Biologia and Institut de Recerca de la Biodiversitat (IRBio), Universitat de Barcelona, Barcelona, Spain

## Abstract

Cytological studies revealed that the number of chromosomes and their organization varies across species. The increasing availability of whole genome sequences of multiple species across specific phylogenies has confirmed and greatly extended these cytological observations. In the Drosophila genus, the ancestral karyotype consists of five rod-like acrocentric chromosomes (Muller elements A to E) and one dot-like chromosome (element F), each exhibiting a generally conserved gene content. Chromosomal fusions and paracentric inversions are thus the major contributors, respectively, to chromosome number variation among species and to gene order variation within chromosomal element. The subobscura cluster of Drosophila consists in three species that retain the genus ancestral karyotype and differ by a reduced number of fixed inversions. Here, we have used cytological information and the *D. guanche* genome sequence to identify and molecularly characterize the breakpoints of inversions that became fixed since the *D. guanche-D. subobscura* split. Our results have led us to propose a modified version of the *D. guanche* cytological map of its X chromosome, and to establish that (i) most inversions became fixed in the *D. subobscura* lineage and (ii) the order in which the four X chromosome overlapping inversions occurred and became fixed.

## Introduction

The last decade witnessed an accelerated development of whole genome sequencing technologies and the concomitant development of the bioinformatics and analytical tools required for genome assembly and annotation as well as for whole genomes evolutionary comparisons both within species and across phylogenies. In many taxa, these comparative analyses unveiled an unprecedented level of structural variation, including duplications, transpositions and inversions^1–5^. In the pre-genomic era, this kind of variation had only been extensively studied in a few genera as its detection did not only require using laborious cytogenetic techniques but it also required that the size of the chromosomes in the species under study was large enough to detect the potential structural differences. In Diptera, the banding pattern of polytene chromosomes provides detailed information on each chromosome organization. Given that structural changes alter this organization, the nature and number of the structural changes that segregate within populations and of those that become fixed in different species can be cytologically inferred through banding pattern comparison. In both the Drosophila and Anopheles genera, the cytological detection of chromosomal inversions segregating in some species propelled their study across space and time (*e.g*., as summarized in Krimbas and Powell^6^ for Drosophila). Moreover, comparison of the detailed cytogenetic maps obtained for genera such as Drosophila and Anopheles allowed the detection of fixed inversions between related species and the subsequent generation of cytological phylogenies^7^.

Comparison of 12 genomes across the Drosophila phylogeny confirmed the cytological observation that paracentric inversions were major contributors to the genus chromosomal evolution^2,3^. It also confirmed, and extended, the cytological observation that some regions had been multiply disrupted by fixed inversions, and that the X chromosome is the fastest evolving chromosome in the genus^3^. Moreover, the molecular identification of inversion breakpoints through genome comparison at short time scales [i.e., either between closely related species such as *Drosophila melanogaster, D. simulans* and *D. yakuba*^8^, or within species^9^] allowed the detailed characterization of inversion breakpoints, which revealed that inversions could originate by the staggered breaks mechanism in addition to the cut-and-paste and ectopic recombination mechanisms.

Both the cytological and genome-based approaches to identify inversions and to finely localize their breakpoints through banding pattern and genome comparison, respectively, have limitations. The most obvious limitation of the cytological approach in species with polytene chromosomes is that imposed by the size of the structural change, as this approach precludes the identification of small inversions. The time elapsed since the divergence of the species under study would constitute a second limitation to identify fixed inversions as chromosomal changes accumulate through time. This accumulation implies that the banding pattern comparison might render the cytological identification of homologous fragments uncertain or even impossible when distantly related species are compared. This uncertainty or impossibility would preclude the identification of the multiple structural changes that are fixed at the long time scale. Even at a shorter time scale, this limitation might differentially affect those chromosomes with a higher rate of chromosomal evolution by paracentric inversions, as it is the case of the X chromosome in Drosophila^3^. Concerning the genome-based approach, the quality of the genomes to be compared would be the major limitation. Indeed, even if the number of species with sequenced genomes is steadily increasing, only a few of the newly generated genome assemblies are composed of super-scaffolds where a relatively reduced number of scaffolds that account for most of each chromosome length are ordered and oriented. These assemblies are especially suitable for the detailed molecular characterization of fixed inversions breakpoints through genome comparison. In contrast, the draft assemblies of the remaining species genomes would be inadequate to address questions on chromosomal evolution, as they would preclude the fine molecular identification of fixed inversions breakpoints.

The subobscura cluster of Drosophila is composed of three species: *D. subobscura*, *D. madeirensis* and *D. guanche*. The former species is widely distributed whereas the other two species are island endemics that originated in Madeira and the Canary Islands, respectively, upon their independent colonization by *D. subobscura*. The karyotype of these species consists of five rod-like acrocentric chromosomes and one dot-like chromosome named A (X), J, U, E, O and dot that correspond to Muller A, D, B, C, E and F elements, respectively. These species structural variation has been extensively studied at the cytological level (as summarized in^10^). Unlike the two island endemic species that are monomorphic at the chromosomal level^11–13^, *D. subobscura* exhibits a rich inversion polymorphism that presents adaptive latitudinal clines in both its original palearctic distribution area and in the newly colonized areas in the west coast of both American subcontinents^14^. The breakpoints of several of the *D. subobscura* polymorphic inversions have been molecularly identified and characterized through chromosome walking, which has revealed that the staggered breaks mechanism that generates duplications in the derived arrangement is the prevalent mechanism originating inversions in this species^15–20^, like it also is in *D. melanogaster*^9,21^.

**Figure 1:**
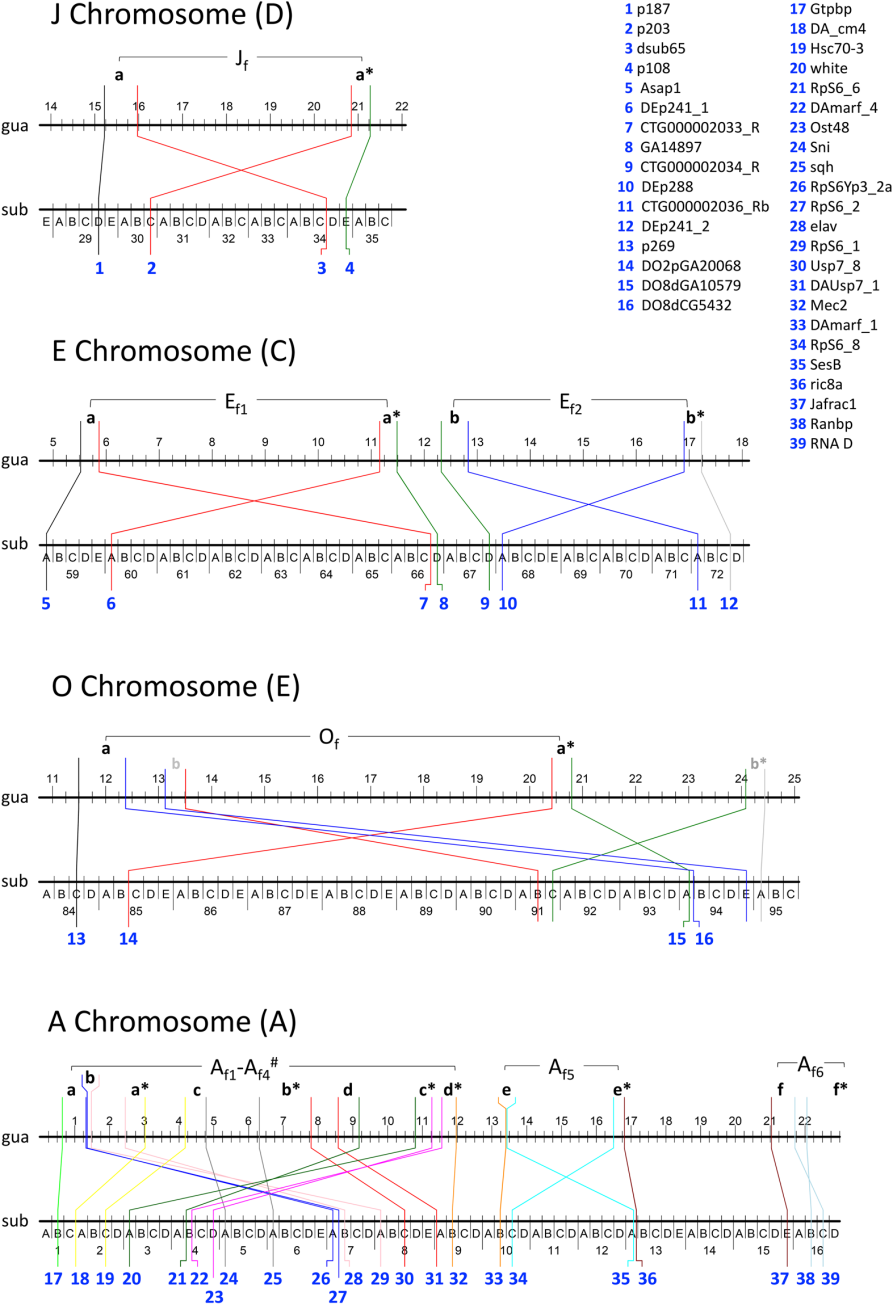
*Drosophila guanche* and *D. subobscura* chromosomes affected by fixed inversions. The localization of inversion breakpoints in *D. subobscura* chromosomes J, E, O and A (Muller elements in parentheses) through their flanking markers is given on a schematic representation of the Kunze-Mühl and Müller^33^ map (not at scale). Localization of each marker sequence in the *D. guanche* genome sequence is represented above on a horizontal line (in Mb units). Fragments delimited by differently colored lines include inversion breakpoints, with the *D. guanche* fragments spanning both breakpoints of a given inversion labeled with the same low-case letter (*e.g*., a, proximal breakpoint; a*, distal breakpoint). The O chromosome of *D. guanche* differs from the O_st_ arrangement of *D. subobscura* by two overlapping inversions, the O_f_ inversion and the *D. subobscura* polymorphic inversion O_3_. Although both inversions are shown, only markers flanking the O_f_ inversion —fixed since the two species divergence— are given. For the four overlapping inversions of the A chromosome (#), fragments labeled a, b, c and d correspond to the breakpoints of inversions A_f1_, A_f2_, A_f3_ and A_f4_, respectively. Markers delimiting inversion breakpoints are labeled 1 to 39.

Comparison of the cytological maps of *D. subobscura* and *D. guanche* had revealed that the *D. guanche* chromosomes differ from the standard arrangement of all chromosomes of *D. subobscura* by 13 inversions^11,13,22^ —six on the A chromosome and seven in the four large acrocentric chromosomes. Ten of these inversions became fixed since the *D. subobscura-D. guanche* split whereas the other three originated and still segregate in *D. subobscura*^10^. Although a high-quality assembly of the *D. guanche* genome has been recently obtained^23^, this is not yet the case for *D. subobscura*, which precludes the identification of inversions breakpoints in both species through genome comparison. In *D. subobscura*, over 500 sequence-based markers have been cytologically mapped, with a small subset also mapped in *D. madeirensis* and *D. guanche*^23,24^. Given the extended collinearity previously detected between *D. subobscura* and *D. guanche* in most autosomal regions and in parts of the A chromosome, we have combined the information provided by the newly generated *D. guanche* genome and by previously mapped markers with known sequence to molecularly identify the breakpoints of the ten inversions —four in autosomes J, E and O and six on the A chromosome (Fig. 1)— that became fixed since the *D. subobscura-D. guanche* split^11,13^. The identification and molecular characterization of these inversions breakpoints will not only allow us to contrast the available, and sometimes differing, cytological information on these inversions but it might provide information on their mechanism of origin. It will additionally allow us to identify in which lineage each inversion occurred and became fixed, and therefore to infer a molecularly based phylogeny of the paracentric inversions underlying the chromosomal evolution of the subobscura cluster.

## Results

### Identification of fixed inversions breakpoints

The relevant cytological and molecular information used to identify the breakpoints of the four autosomal and six A chromosome inversions fixed since the divergence of *D. subobscura* and *D. guanche* is summarized in Fig. 1. These inversions have been named J_f,_ E_f1_, E_f2_, O_f_ and A_f1_ to A_f6_, according to the chromosome affected and numbered in each chromosome according to their centromere-proximal position. Our strategy was based on (i) the cytological localization of 335 markers with known sequence that had been previously mapped on the J, E, O and A chromosomes of *D. subobscura*, as summarized in Puerma *et al*.^23^, and (ii) the localization of these markers in the recently assembled *D. guanche* genome sequence^23^. Comparison of these datasets revealed discontinuities in markers order —two for each of the independent inversions J_f,_ E_f1_, E_f2_, O_f_ and A_f5_ and overlapping inversions A_f1_ to A_f4_, and only one for inversion A_f6_, as it is a terminal inversion (Fig. 1). These discontinuities allowed us to identify those markers flanking the breakpoints of each inversion (Fig. 1) and therefore to delimit a large region spanning each breakpoint in *D. guanche*, with the only exception of the distal breakpoint of inversion A_f6_.

For each inversion, the large region spanning a breakpoint in *D. guanche* was initially compared to draft2 of the *D. subobscura* genome (Barcelona Subobscura Initiative [BSI]) in order to more narrowly delimit the breakpoint region. For breakpoints of the four autosomal inversions, this comparison allowed the identification of a rather short *D. guanche* fragment that spanned the breakpoint. For the A chromosome inversions, this approach was generally unsuccessful, which led us to initiate chromosomal walks from both ends of the large regions that in *D. guanche* spanned the breakpoints. For both autosomal and A chromosome breakpoint regions, probes were designed and amplified on the *D. guanche* genome and they were subsequently *in situ* hybridized on polytene chromosomes of *D. subobscura* until one of them gave two signals approximately at the locations expected according to either Moltó *et al*.^11^ or Brehm and Krimbas^13^, indicating that the probe spanned the breakpoint. In the case of autosomal inversions, one or a few probes were required for breakpoint identification whereas a higher number of probes was generally needed to narrow down and eventually cross each A chromosome inversion breakpoint. The final probes spanning the breakpoints of the four autosomal inversions and the A chromosome inversions A_f2_ to A_f5_ were additionally *in situ* hybridized on polytene chromosomes of *D. guanche* where they gave one signal at or rather close to the expected band (Supplementary Figs S1 to S8; Table 1). The adequate combination of primers designed on *D. guanche* was used to amplify the fragments that in *D. subobscura* spanned each inversion breakpoint and only in a few cases, new primers had to be designed on draft2 of the *D. subobscura* genome (BSI). When these fragments were hybridized on polytene chromosomes of both species, they generally gave a single signal on *D. subobscura* chromosomes and two signals on *D. guanche* chromosomes at the same bands that fragments spanning the *D. guanche* breakpoints had (Supplementary Figs S1 to S8). The *D. subobscura* fragments spanning the breakpoints were subsequently sequenced, annotated and compared to the *D. guanche* genome, which allowed us to delimit and characterize each breakpoint (see below).

**Table 1:** Proposed cytological boundaries of inversions fixed between *D. subobscura* and *D. guanche*.

Inversion	Moltó *et al*. 1987^a^	Brehm and Krimbas 1990^a^	Present work
A_f1_	2A/B-7C/D	1C/2A-6E/7A	1C/2A-7B
A_f2_	6E/D-7D/8A	**1C/6E**-8A/8B	7A-8A
A_f3_	**2D/8D**-4D/4C	2D/3A-4D/5A	2D/3A-4D
A_f4_	2D/3A-8D/E	4A/B-8E/9A	4B-9A
A_f5_	10C-13A/B	10C-13A/B	10C-13A
A_f6_	16BCD	16BCD	16BCD
J_f_	30A-34E	30A-34E	30A-34E
E_f1_	59D-66C/D	59D-66C/D	59D-66D
E_f2_	67C/D-72B/C	67C-72B/C	67D-72B
O_f_	84D/85A-93D/94A	84D/85A-93D/94A	85A/B-94A

^a^Numbers in bold indicate reused breakpoints.

The breakpoints of inversions A_f1_ and A_f6_ could not be identified but they were narrowed down to rather short regions. Indeed, breakpoints of inversion A_f1_ were narrowed down to ~44-kb (flanked by genes GA14783 and GA13678) and ~33-kb long (flanked by genes GA17070 and GA15499) regions of the assembled A chromosome of *D. guanche*, and the proximal breakpoint of inversion A_f6_ to an ~20-kb long region (flanked by genes GA22805 and GA24354). Our failure to design new probes on *D. guanche* in each of the three intervening regions was due either to the discontinuity between two neighboring scaffolds in the *D. guanche* assembled A chromosome (proximal A_f1_ breakpoint) or because of the abundance of transposable elements and other repetitive sequences (two other breakpoints).

**Figure 2:**
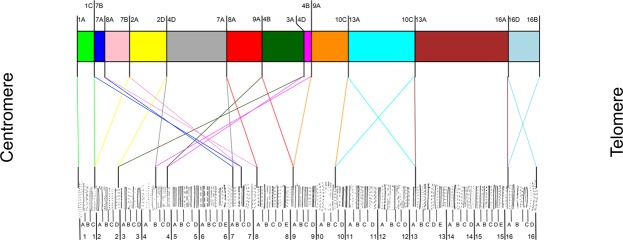
Newly proposed cytological map of the *D. guanche* A chromosome. Colored boxes represent conserved blocks between *D. guanche* and *D. subobscura* based on the localization of breakpoints in the recently assembled *D. guanche* genome (Puerma *et al*.^23^). Sections delimiting each conserved block, given in their upper part, were inferred from the localization in the Kunze-Mühl and Müller^33^ map of *D. subobscura* of the fragments spanning the six fixed inversions breakpoints in *D. guanche* (Puerma *et al*.^23^). Differently colored lines stemming from a given breakpoint in the *D. guanche* map indicate each of its flanking regions and their localization in the Kunze-Mühl and Müller^33^ map of *D. subobscura*, as revealed by *in situ* hybridization.

For the A chromosome inversions, results of the *in situ* hybridizations on *D. subobscura* polytene chromosomes revealed some discrepancies not only concerning the number of breakpoints but also their cytological localization relative to those previously proposed^11,13^ (Table 1). This led us to propose a modified version of the cytological map of the *D*. *guanche* A chromosome (Table 1 and Fig. 2). The major difference between the here proposed map and previous proposals stems from the more limited resolution of the classic cytological approach. Indeed, as opposed to the eight breakpoints here identified for the four overlapping inversions in segment I of the A chromosome, those studies could only identify seven breakpoints, which implied reuse of one of the breakpoints (Table 1).

### Characterization of fixed inversions breakpoints

**Figure 3:**
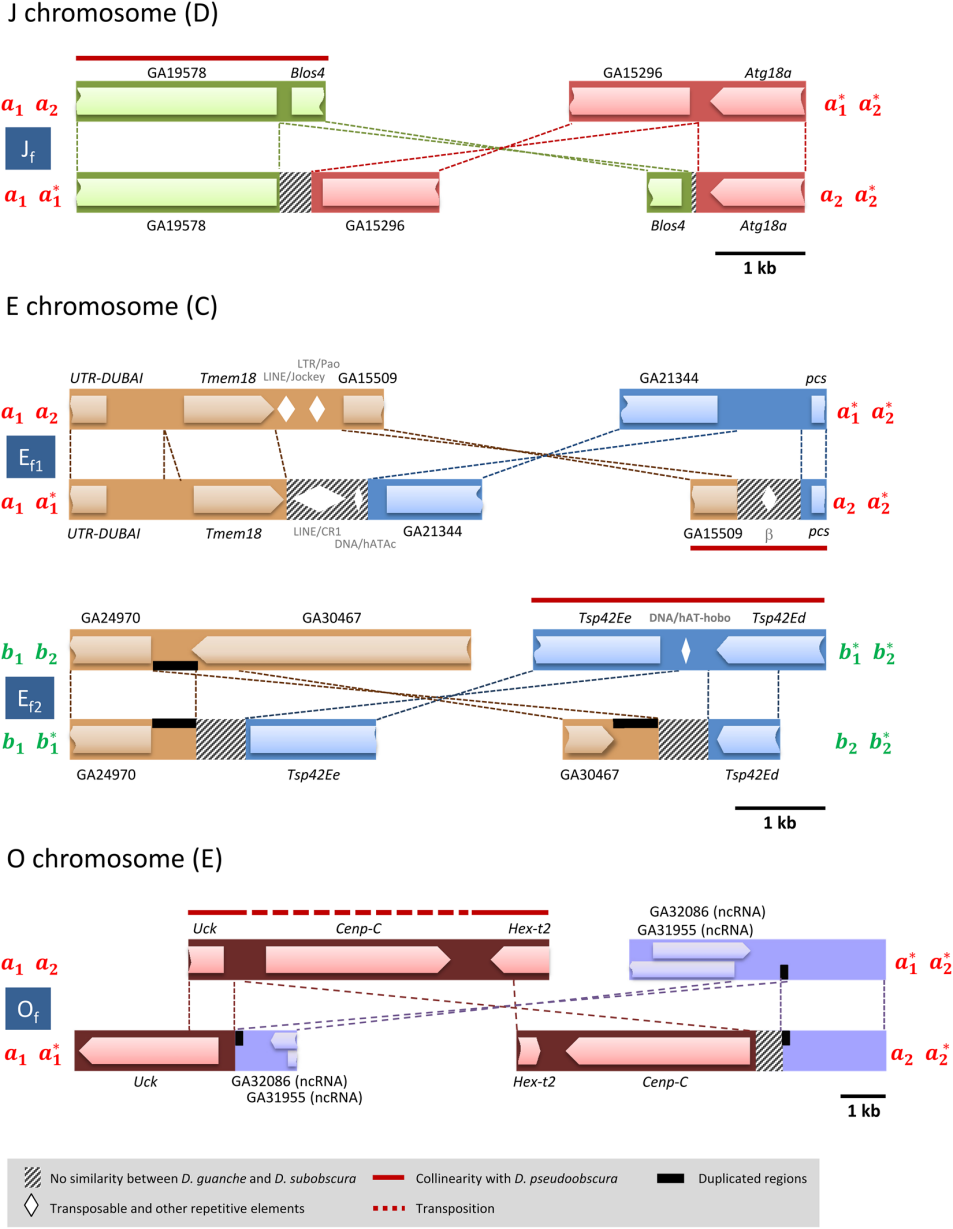
Functional annotation of autosomal inversion breakpoints. Schematic representation of the sequenced and annotated breakpoint regions corresponding to the four autosomal inversions fixed since the *D. subobscura-D. guanche* split. *D. guanche*, above; *D. subobscura*, below. Dashed lines between chromosomal arrangements indicate the limits and orientation of homologous regions. Arrowed bars represent annotated coding regions whereas rhombuses represent annotated transposable elements and other repetitive sequences. Thick dark red lines above or below a particular breakpoint region indicate its collinearity relative to *D. pseudoobscura*.

**Figure 4:**
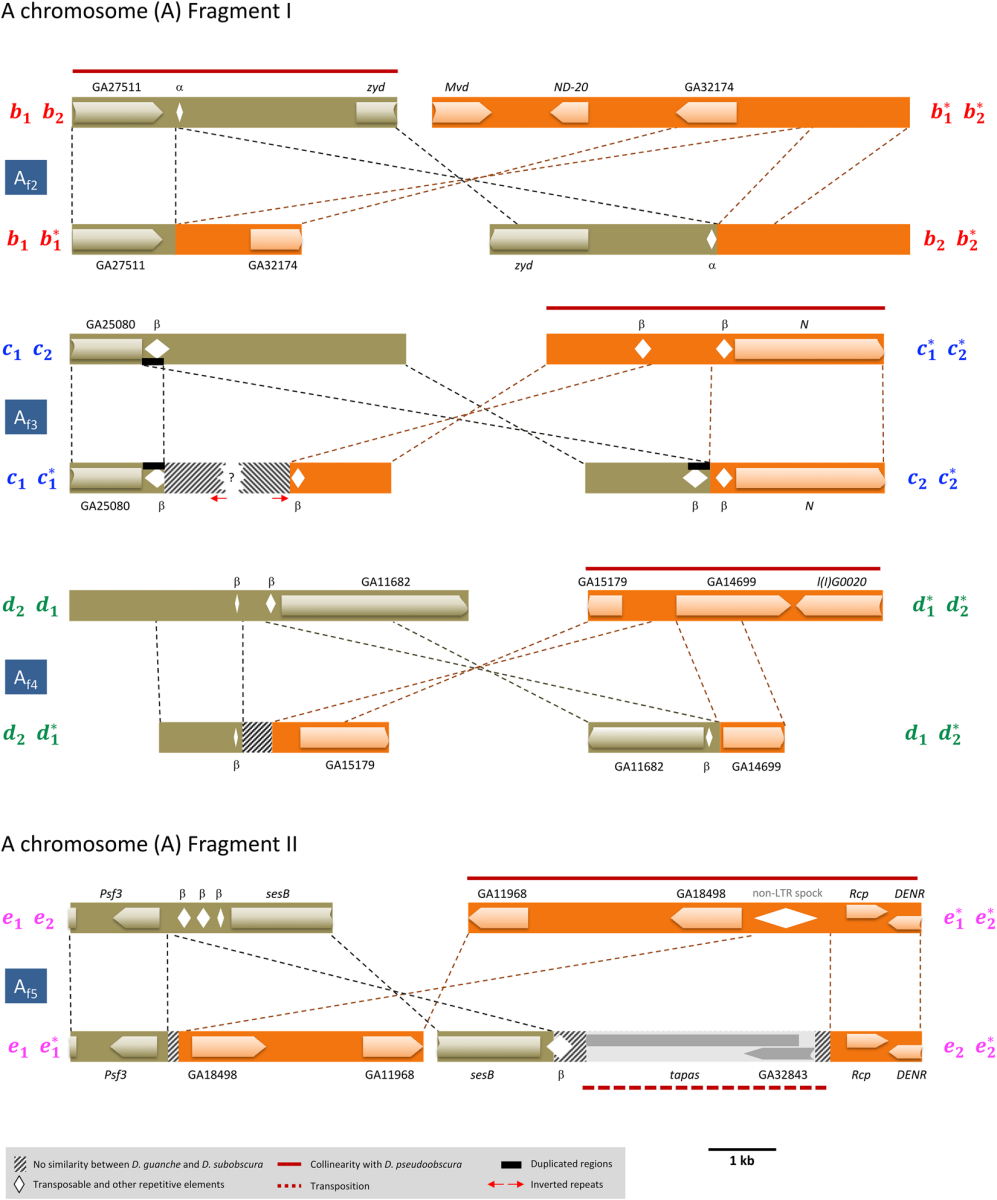
Functional annotation of A chromosome inversion breakpoints. Schematic representation of the annotated breakpoint regions corresponding to X chromosome inversions A_f2_ to A_f5_ that originated and became fixed since the *D. subobscura-D. guanche* split. *D. guanche*, above; *D. subobscura*, below. Dashed lines between chromosomal arrangements indicate the limits and orientation of homologous regions. Arrowed bars represent annotated coding regions whereas rhombuses represent annotated transposable elements and other repetitive sequences. Thick dark red lines above a particular breakpoint region indicate its collinearity relative to *D. pseudoobscura*.?, missing information.

Figures 3 and 4 show the functional annotation of the breakpoint regions of the four autosomal inversions and the A chromosome inversions A_f2_ to A_f5_ here studied, respectively. It is worth noting that none of the breakpoints disrupt genic regions even though according to the *D. pseudoobscura* annotation, the proximal *D. guanche* breakpoints of inversions J_f_ and E_f1_ would disrupt the 5′ UTRs of genes GA19578 and GA15509, respectively.

Comparison of the fragments spanning the *D. guanche* and *D. subobscura* breakpoints of the four autosomal and A chromosome A_f2_ and A_f5_ fixed inversions with the *D. pseudoobscura* genome allowed us to establish which of the two arrangements of each inversion exhibited the ancestral state. Indeed, the *D. guanche* fragments spanning the proximal breakpoints of inversions J_f_, O_f_ and A_f2_, and the distal breakpoints of inversions E_f2_, A_f3_, A_f4_ and A_f5_ are collinear or partially collinear with *D. pseudoobscura* (Figs 3 and 4). The presence of gene *Cenp-C* in both the *D. guanche* and *D. subobscura* breakpoints of inversion O_f_ reflects an intra-chromosomal transposition that occurred in these species ancestor. In contrast, the presence of the overlapping genes *tapas* and GA32843 in the distal *D. subobscura* breakpoint of inversion A_f5_ would reflect an inter-chromosomal transposition that occurred in *D. subobscura* after the *D. guanche* split. Concerning inversion E_f1_, the *D. subobscura* arrangement is the ancestral arrangement for the affected region as revealed by the collinearity relative to *D. pseudoobscura* exhibited by its distal breakpoint region (Fig. 3).

In inversions E_f2_, O_f_ and A_f3_, the presence in both *D. subobscura* breakpoints of a duplicated fragment that was present in only one of their *D. guanche* breakpoints (Figs 3 and 4) is consistent with the three inversions having originated by the staggered-breaks mechanism from the *D. guanche* ancestral arrangement. Concerning the remaining inversions, there is no indication that they might have originated through ectopic recombination given the present absence of repeat motifs in inverted orientation at the two breakpoints of any of them in both *D. guanche* and *D. subobscura* (Figs 3 and 4).

## Discussion

In the subobscura cluster of Drosophila, inversions fixed differentially between any of its three species pairs were identified either through banding pattern comparison between species^11,25–27^ or through the observation of polytene chromosomes in hybrids between *D. madeirensis* and either *D. subobscura* or *D. guanche*^12,13,22^. Concerning the more distantly related species —*D. subobscura* and *D. guanche*—, the number of inversions inferred through banding pattern comparison was the same in the different studies for both their autosomes and A chromosome^11,13^. There were, however, some discrepancies concerning the breakpoint assignment of some of the multiple overlapping inversions that had become sequentially fixed in segment I of the A chromosome since the *D. subobscura-D. guanche* split, and therefore also in the putative order in which these inversions occurred^11,13^. Moreover, comparison of the banding pattern of the three species of the subobscura cluster and those of six other species of the obscura group did not allow the establishment of how the different members of the subobscura cluster are related to the other six species, except for the J and E chromosomes, for which the subobscura cluster ancestor would have the arrangement presently found in *D. guanche*^27^.

Concerning the three species of the subobscura cluster, polytene chromosomes in hybrids between *D. madeirensis* and either *D. subobscura* or *D. guanche*^12,13,22^ revealed that at the cytological level (i) *D. madeirensis* only differed from the standard arrangement of the *D. subobscura* chromosomes by two A chromosome inversions and three autosomal inversions^12,22^, (ii) for the A chromosome, the differences exhibited by *D. madeirensis* relative to *D. subobscura* are shared by *D. guanche*^13^, and (iii) the most centromere-proximal A chromosome inversion present in *D. madeirensis* differed from the *D. subobscura* polymorphic A_1_ inversion^12^.

**Figure 5:**
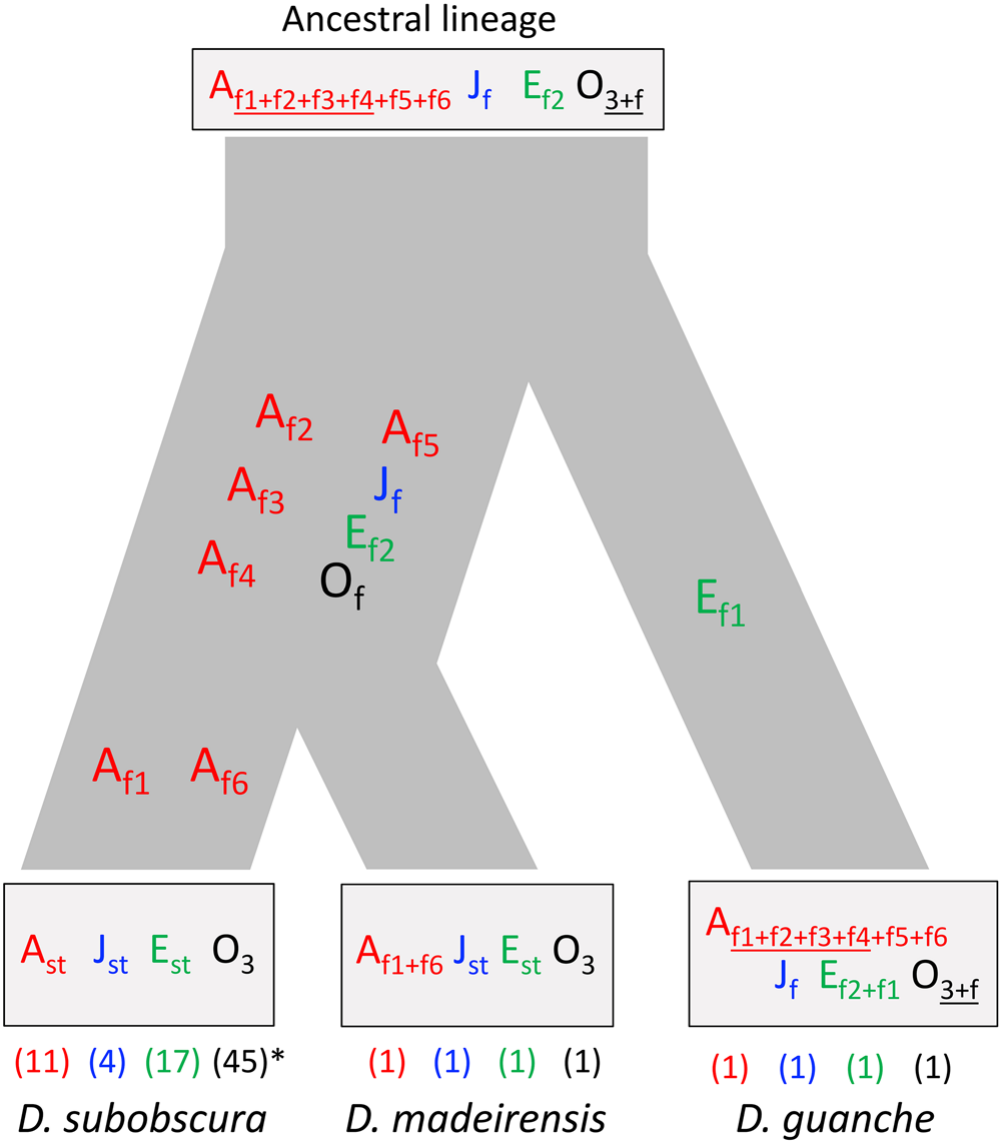
Distribution across the subobscura cluster phylogeny of the ten fixed inversions since the *D. subobscura-D. guanche* split. The ancestral arrangement of chromosomes J, E, O and A (X), as inferred in the present study, is given in the cluster ancestor. Inversions that became fixed in these chromosomes are presented in the different branches of the phylogeny. The arrangement for each chromosome upon the fixation of the 10 inversions is given for each lineage, with numbers in parentheses referring to presently polymorphic arrangements as a result of inversions that originated thereafter in the four affected chromosomes. *Inversion O_3_ is currently polymorphic in *D. subobscura* and always associated with inversion O_4_.

The molecular characterization of the breakpoints of eight of the ten inversions that became fixed since the divergence of *D. subobscura* and *D. guanche* and their comparison with the *D. pseudoobscura* genome has allowed us to detect collinearity and therefore to infer which was the chromosomal arrangement for each of these inversions that was present in the ancestor of the subobscura cluster. Our results would support that the *D. subobscura* population that colonized the Canary Islands archipelago had the same chromosomal arrangement than *D. guanche* presently has in seven of the eight regions affected by the fixed inversions with breakpoints here characterized, being the region affected by inversion E_f1_ the only exception (Fig. 5). Moreover, previous cytological studies had revealed that *D. guanche* and *D. madeirensis* share the same arrangement at the regions affected by inversions A_f1_ and A_f6_^22^. It can be therefore concluded that i) only inversion E_f1_ would have occurred and become fixed upon the colonization of the Canary Islands by *D. subobscura* (*i.e*., in *D. guanche*), and ii) the chromosomal arrangement at the remaining nine regions of *D. guanche* would be a relict of the arrangements present at the ancestor of the subobscura cluster (Fig. 5). It can be, moreover, inferred that seven of these inversions —autosomal inversions J_f_, E_f2_ and O_f_ as well as A chromosome inversions A_f2_ to A_f5_— became fixed in *D. subobscura* after this species colonized the Canary Islands but prior to the colonization of the Madeira island (*i.e*., between 1.8 and 0.6 million years ago according to previous estimates of the corresponding species divergence times^28^) whereas A chromosome inversions A_f1_ and A_f6_ would have become fixed thereafter in *D. subobscura*. Concerning the E chromosome, the present inference stands in contrast with the ancestral character of the E_f1+f2_ arrangement inferred from the banding pattern comparison of *D. guanche* and other six species of the obscura group^27^, which might be due to the limited resolution of banding pattern comparison when distantly related species are compared. The much higher number of inversions fixed in the broadly distributed *D. subobscura* than in the island endemic *D. guanche* would be at odds with expectations if these inversions had not affected, or slightly affected, the fitness of their bearers. It should be however considered that the continental species possibly experienced more environmental challenges than the island species, and that the adaptive character of at least some of the inversions that emerged in *D. subobscura* might have contributed to cope with these challenges, and consequently, led them to fixation. The putative selective advantage of any of these inversions could be due to the structural change itself^29–31^ or to the particular variant of one or more of the genes included in the region affected by the structural mutation.

Concerning inversion A_f1_ and the *D. subobscura* A_1_ polymorphic inversion, the breakpoints of neither inversion could be identified^20^ (present work) but they were narrowed down to differently sized regions of the *D. guanche* assembled chromosome (see Results section). The localization of the proximal and distal A_f1_ breakpoint regions in *D. guanche* differs by at least 880 and 790 kb, respectively, from that inferred through BLAST search of the corresponding regions of the *D. subobscura* A_1_ inversion^20^, which corroborates the cytological results obtained in *D. subobscura-D. madeirensis* hybrids^12,22^.

**Figure 6:**
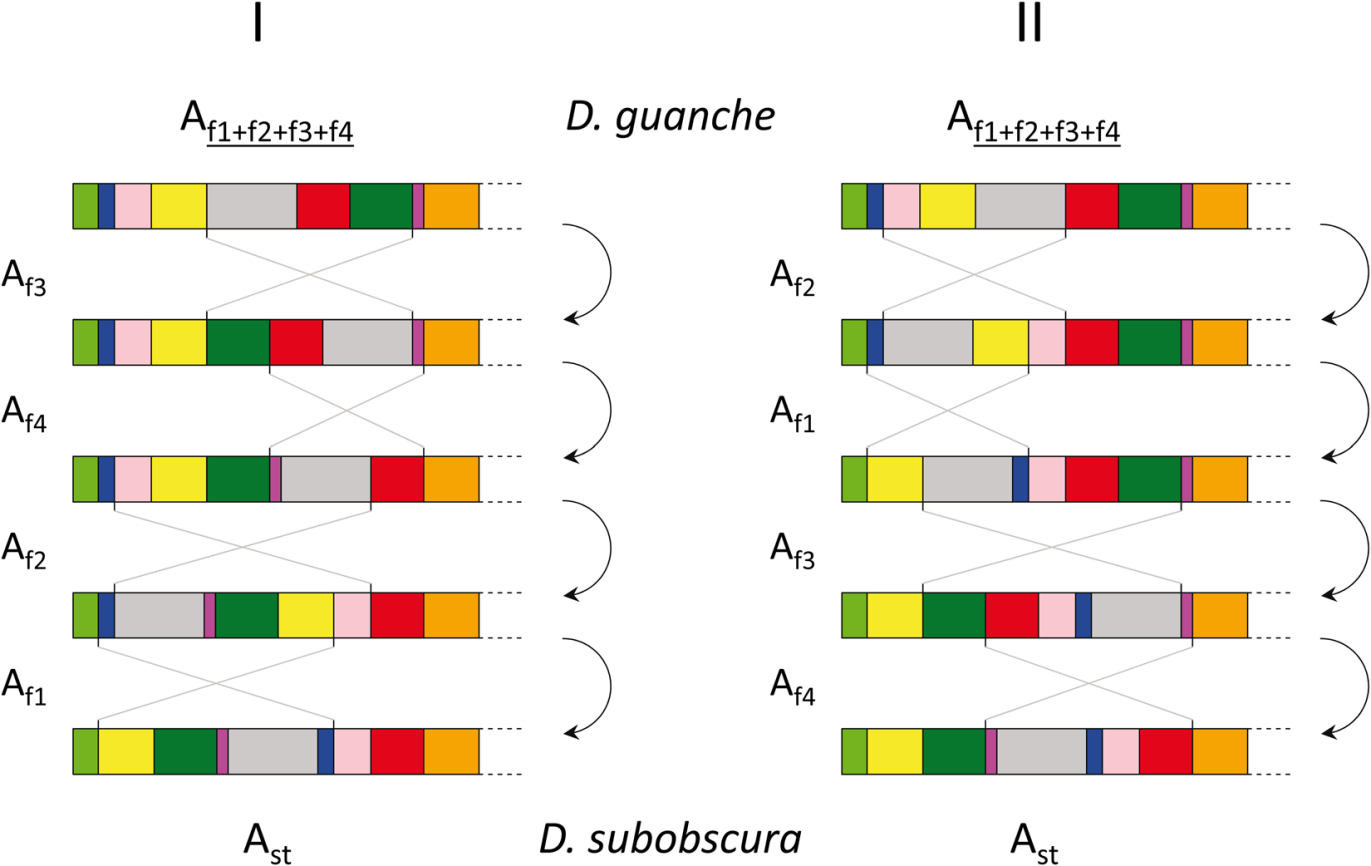
Sequential order of occurrence and fixation in the *D. subobscura* lineage of the four A chromosome overlapping inversions. Schematic representation of the order established in the present study (I) and its discarded alternative (II) for the sequential occurrence and fixation of inversions A_f1_ to A_f4_ of the A chromosome in the *D. subobscura* lineage after its split from *D. guanche* (see text). Horizontal bars with differently colored boxes represent the different chromosomal arrangements, with the ancestral order (now only present in *D. guanche*) in the upmost part of the figure and the *D. subobscura* A_st_ order in its lowest part. Boxes colored as in Fig. 2 reflect conserved blocks relative to *D. subobscura*. Pairs of crossed lines between arrangements represent the regions affected by each of the four inversions. Arrows connecting the different chromosomal arrangements represent the sequential accumulation of inversions from the ancestral *D. guanche* arrangement (see text).

Upon establishing the ancestral character of the *D. guanche* arrangement for segment I of the A chromosome, we aimed at inferring the order in which its four overlapping inversions occurred and became fixed in the *D. subobscura* lineage. Our identification of both breakpoints of each of these four inversions in *D. guanche* has resulted in a cytological map with increased resolution, which has allowed us to accomplish our goal. Figure 6 represents the two possible orders based on our results in which the four inversions might have accumulated and led from the A chromosome arrangement present in the subobscura cluster ancestor —now only present in *D. guanche* (Fig. 2)— to the A_st_ segment I arrangement of *D. subobscura*. Given that segment I of *D. madeirensis* only differs from the *D. subobscura* A_st_ arrangement by the A_f1_ inversion, it can be inferred that this inversion that occurred upon the *D. madeirensis*-*D. subobscura* split was the last one to occur^13^. This allows us to unambiguously establish that the order in which the four inversions occurred is the following: A_f3_, A_f4_, A_f2_ and A_f1_ (Fig. 6-I).

Concerning the eight fixed inversions with breakpoints here molecularly characterized, our results are consistent with three of them having originated by the staggered-breaks mechanism whereas they are not informative on the mechanism involved in the origin of the other five (Figs 3 and 4). Our results would seem to be in contrast with those previously obtained for nine polymorphic inversions of *D. subobscura*^15–20^ where the staggered-breaks mechanism was considered the most common (proposed in eight out of nine inversions with breakpoints molecularly characterized). It should be however noted that the point and structural mutations that accumulate through time at the breakpoint regions tend to erode the signals left by the different originating mechanisms. This erosion would render polymorphic inversions more adequate than fixed inversions to establish the role played by the different originating mechanisms given the differential time scale of fixed and polymorphic inversions occurrence. Finally, our analysis of the *D. guanche* and *D. subobscura* breakpoint regions revealed two transpositions. This observation together with the loss of collinearity relative to *D. pseudoobscura* detected at one of the two breakpoint regions of each ancestral arrangement indicates that additional structural changes have occurred in the vicinity of inversion breakpoints, as previously observed at the extended breakpoint regions of some *D. subobscura* polymorphic inversions^16–18^.

In summary, we have identified and molecularly characterized the breakpoints of eight of the ten chromosomal inversions —four autosomal and six sex-linked— that became fixed since the *D. subobscura*-*D. guanche* split. The breakpoints of the other two fixed inversions could only be narrowly delimited. Based on our molecular and cytological results, we propose a modified version of the cytological map of the *D. guanche* A chromosome. The molecular information here obtained together with the previous cytological results have allowed us to establish that in nine of the ten regions affected by fixed inversions, *D. guanche* can be considered a relict exhibiting the chromosomal arrangements present at the ancestor of the subobscura cluster. This information has resulted in one of the first chromosomal phylogenies based on the comparison of inversion breakpoint sequences, and constitutes therefore an important contribution to advance our knowledge on chromosomal evolution. Finally, we have been able to unambiguously establish the order in which the four A chromosome overlapping inversions occurred and became fixed.

## Materials and Methods

One *Drosophila guanche* (GI_16) isogenic strain and four *D. subobscura* (*ch cu*, OF28, OF40 and FO31c) isogenic strains were used in the present study. The *D. guanche* GI_16 strain is homokaryotypic for all chromosomes^32^ whereas the *D. subobscura* strains are either homokaryotypic for the five acrocentric chromosomes (*ch cu* for A_st_, J_st_, U_st_, E_st_ and O_3+4_; and OF28 for A_st_, J_1_, U_1+2+8_, E_st_ and O_st_) or for a subset of them (OF40 for A_st_, J_1_, E_st_ and O_3+4+8_; and FO31c for J_st_, U_st_, E_st_ and O_3+4+2_). Isogenic lines were obtained by at least 12 generations of brother-sister sib-mating^16,19^.

The sequences of 335 molecular markers with known cytological information in *D. subobscura* were used to identify their *D. guanche* homologs through BLAST search. Comparison of these datasets allowed us to delimit a large region spanning each inversion breakpoint in *D. guanche*. In order to identify each inversion breakpoint, the sequence of each *D. guanche* large region was thereafter either i) compared to draft2 of the *D. subobscura* genome (BSI) to further delimit the breakpoint region, or ii) used to initiate chromosomal walks. Oligonucleotides to amplify additional probes were designed on the *D. guanche* genome^23^ and in a few cases on draft2 of the *D. subobscura* genome (BSI). Probes were amplified by PCR using TaKaRa DNA polymerase (Takara Bio Inc) and genomic DNA from either the *D. guanche* GI_16 or the *D. subobscura ch cu* strains [using the Puregen Cell kit B (Qiagen)]. Upon their Biotin-16-dUTP (Roche) labeling, they were *in situ* hybridized on polytene chromosomes of one of the four *D. subobscura* strains (*ch cu*, OF28, OF40 and FO31c) whereas those crossing each breakpoint were also hybridized on the *D. guanche* GI_16 strain. Hybridization signals were subsequently located on the *D. subobscura* cytological map^33^. All steps of the *in situ* hybridization procedure were performed as described in Montgomery *et al*.^34^ with minor modifications. Digital images at a 400 magnification were obtained using a Leica DFC290 camera mounted on a phase contrast Axioskop 2 Zeiss microscope.

Fragments spanning the breakpoints in *D. subobscura* were PCR amplified using DNA from the corresponding strain using TaKaRa DNA polymerase (Takara Bio Inc) and oligonucleotides anchored at each breakpoint flanking regions. The amplified fragments were sequenced using primer walking whenever necessary. Amplicons were purified with MultiScreen PCR plates (Millipore) prior to their sequencing with the ABI PRISM version 3.2 cycle sequencing kit. Sequencing products were separated on an ABI PRISM 3730 sequencer. Sequences were assembled using the DNASTAR package (Burland 2000). The *D. subobscura* sequences newly obtained have been deposited in the European Nucleotide Archive (ENA) under project number PRJEB27938.

## Supplementary information

Supplementary Material

## Data Availability

The *D. subobscura* sequences newly obtained have been deposited in the European Nucleotide Archive (ENA) under project number PRJEB27938.
