# Supplementary material for: The molecular characterization of fixed inversions breakpoints unveils the ancestral character of the Drosophila guanche chromosomal arrangements
Source: Sci Rep. 2019 Feb 8;9:1706. doi: 10.1038/s41598-018-37121-5 (PMC6368638; doi:10.1038/s41598-018-37121-5)
Supplement: Supplementary file 1 — Supplementary Material [file 41598_2018_37121_MOESM1_ESM.pdf]

Supplementary material

The molecular characterization of fixed inversions breakpoints unveils the ancestral character of *Drosophila guanche* chromosomal arrangements

Dorcas J. Orengo*, Eva Puerma*, Montserrat Aguadé#

* These authors contributed equally to this work

Author for Correspondence: Montserrat Aguadé; Departament de Genètica, Microbiologia i Estadística, Facultat de Biologia and Institut de Recerca de la Biodiversitat (IRBio), Universitat de Barcelona, Barcelona, Spain, e-mail: maguade@ub.edu

Figure S1. *In situ* hybridization results of the J_f inversion breakpoints probes in *D. guanche* and *D. subobscura* 1

Figure S2. *In situ* hybridization results of the E_{f1} inversion breakpoints probes in *D. guanche* and *D. subobscura* 2

Figure S3. *In situ* hybridization results of the E_{f2} inversion breakpoints probes in *D. guanche* and *D. subobscura* 3

Figure S4. *In situ* hybridization results of the O_f inversion breakpoints probes in *D. guanche* and *D. subobscura* 4

Figure S5. *In situ* hybridization results of the A_{f2} inversion breakpoints probes in *D. guanche* and *D. subobscura* 5

Figure S6. *In situ* hybridization results of the A_{f3} inversion breakpoints probes in *D. guanche* and *D. subobscura* 6

Figure S7. *In situ* hybridization results of the A_{f4} inversion breakpoints probes in *D. guanche* and *D. subobscura* 7

Figure S8. *In situ* hybridization results of the A_{f5} inversion breakpoints probes in *D. guanche* and *D. subobscura* 8

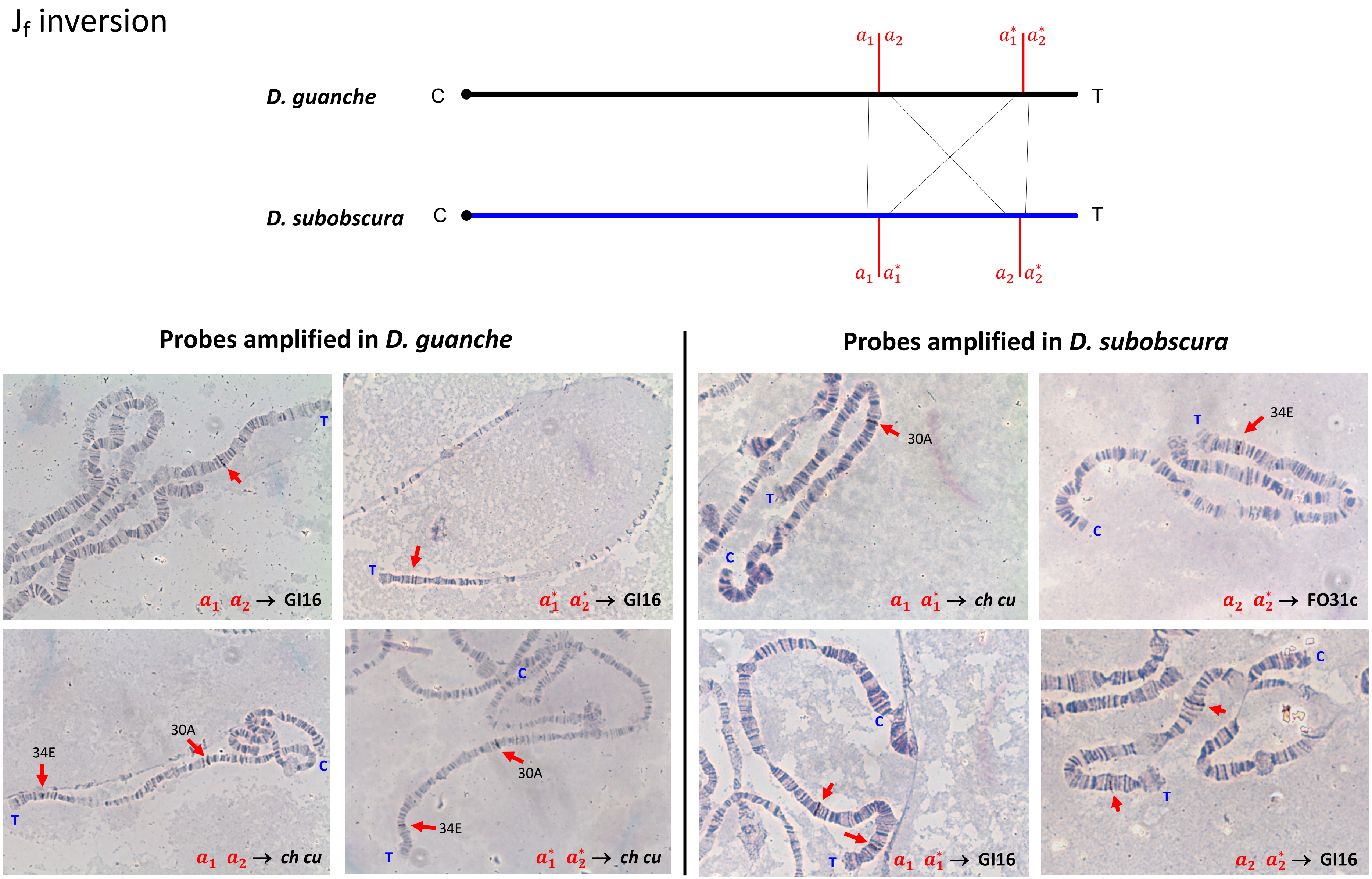

Supplementary Figure S1. ***In situ*** **hybridization results of the J_f inversion breakpoints probes in** ***D. guanche*** **and** ***D. subobscura*****.** Upper part, schematic representation of the J_f inversion using the same notation for breakpoint regions than in Figure 1. Lower part, results of the *in situ* hybridizations performed on both *D. guanche* (GI_16) and *D. subobscura* (*ch cu* or FO31c) polytene chromosomes. Hybridization signals at the inversion breakpoints are marked with red arrows. Their cytological location is indicated according to the Kunze-Mühl and Müller (1958) map of *D. subobscura*. C, centromere; T, telomere.

E_{f1} inversion

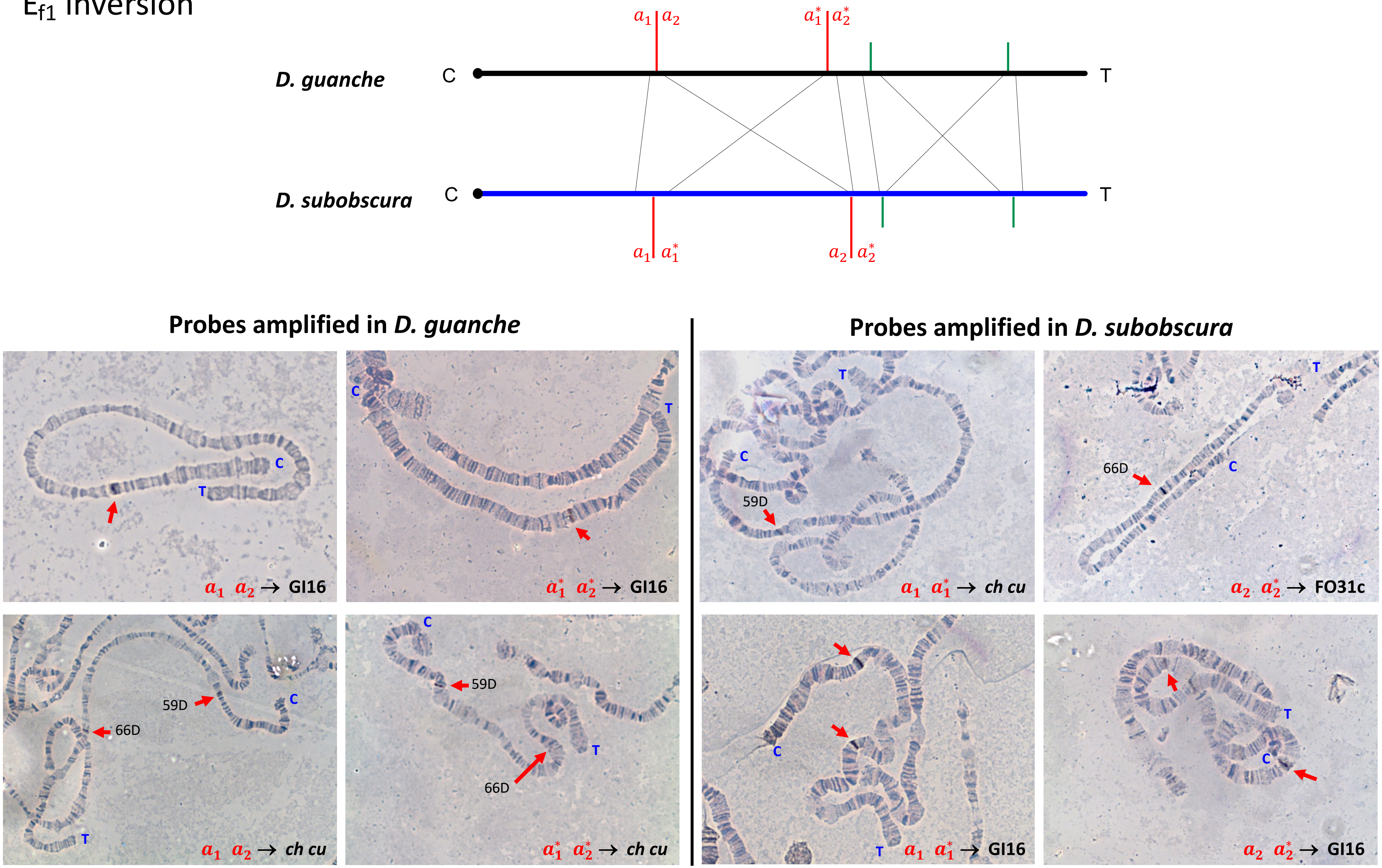

Supplementary Figure S2. ***In situ*** **hybridization results of the E_{f1} inversion breakpoints probes in** ***D. guanche*** **and** ***D. subobscura*****.** Upper part, schematic representation of the E_{f1} inversion using the same notation for breakpoint regions than in Figure 1. Lower part, results of the *in situ* hybridizations performed on both *D. guanche* (GI_16) and *D. subobscura* (*ch cu* or FO31c) polytene chromosomes. Hybridization signals at the inversion breakpoints are marked with red arrows. Their cytological location is indicated according to the Kunze-Mühl and Müller (1958) map of *D. subobscura*. C, centromere; T, telomere.

E_{f2} inversion

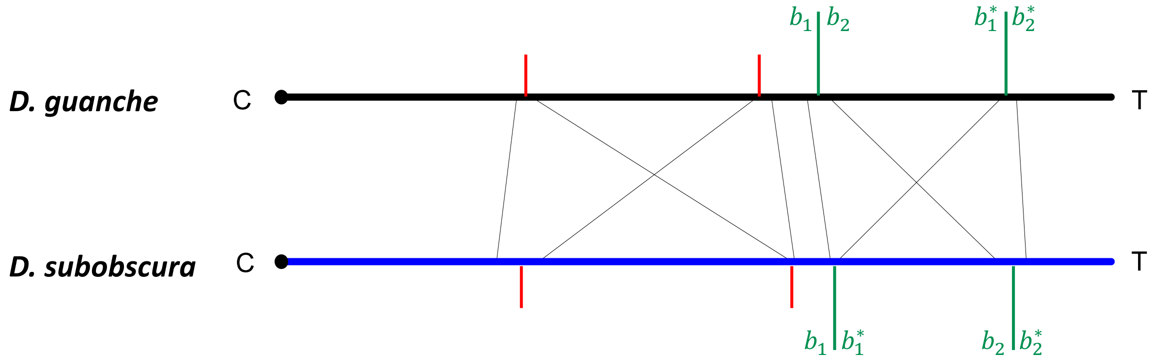

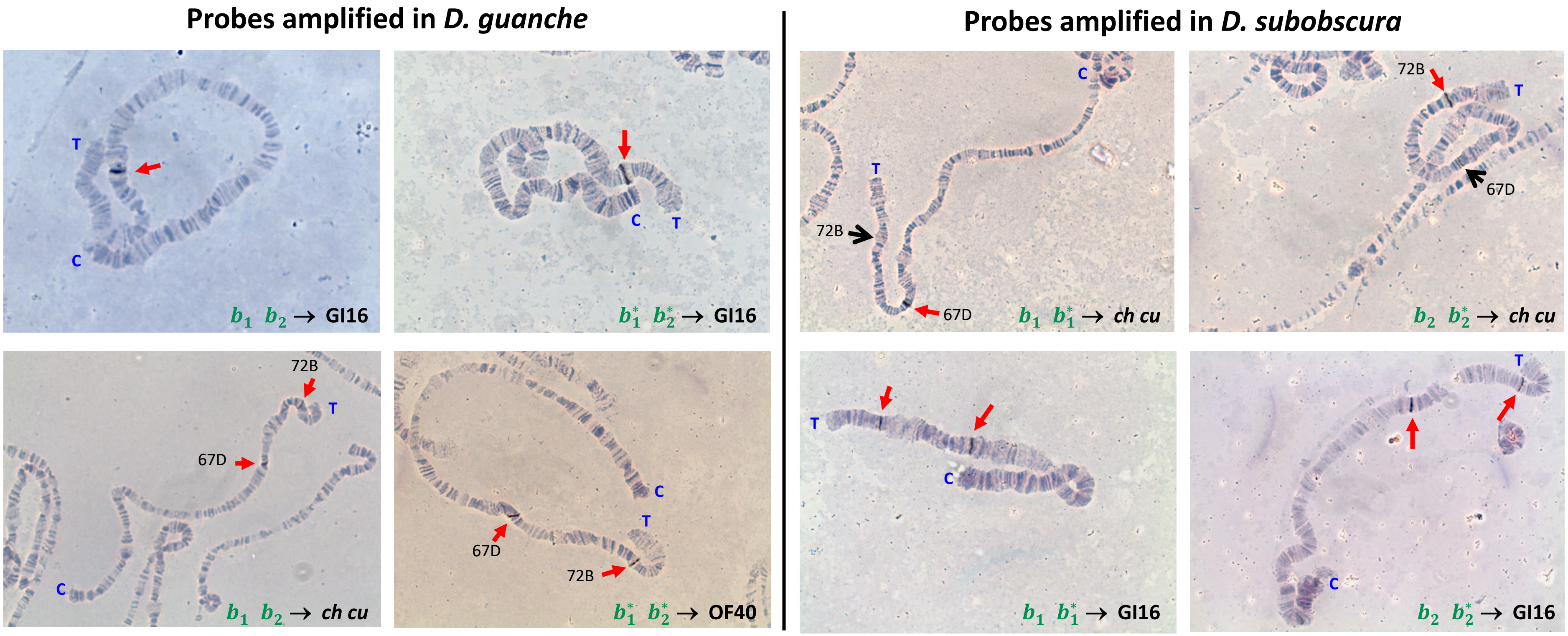

Supplementary Figure S3. *In situ* hybridization results of the E_{f2} inversion breakpoints probes in *D. guanche* and *D. subobscura*. Upper part, schematic representation of the E_{f2} inversion using the same notation for breakpoint regions than in Figure 1. Lower part, results of the *in situ* hybridizations performed on both *D. guanche* (GI_16) and *D. subobscura* (*ch cu* or OF40) polytene chromosomes. Hybridization signals at the inversion breakpoints are marked with red arrows, secondary signal are marked with black arrows. Their cytological location is indicated according to the Kunze-Mühl and Müller (1958) map of *D. subobscura*. C, centromere; T, telomere.

O_f inversion

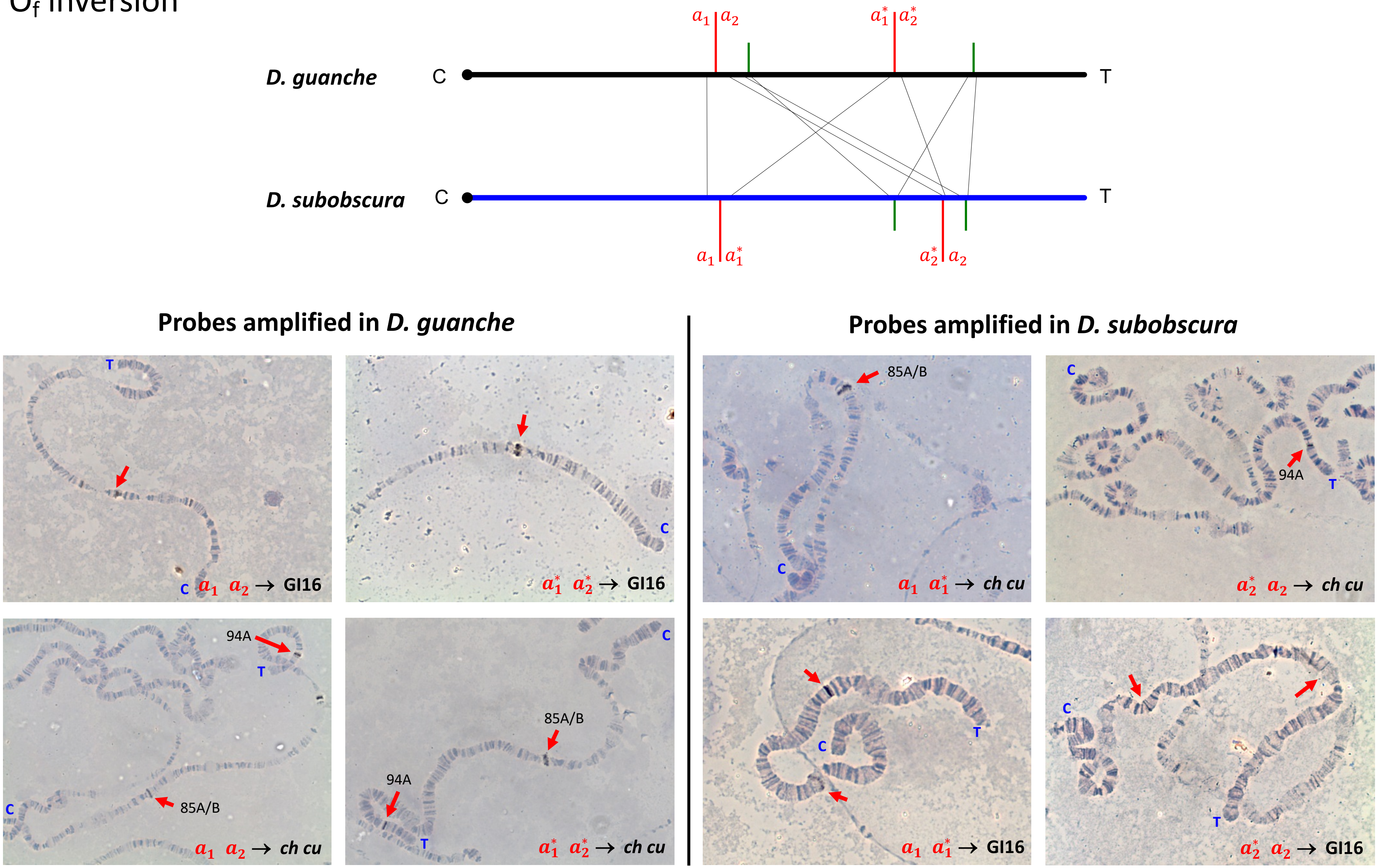

Supplementary Figure S4. *In situ* hybridization results of the O_f inversion breakpoints probes in *D. guanche* and *D. subobscura*. Upper part, schematic representation of the O_f inversion using the same notation for breakpoint regions than in Figure 1. Lower part, results of the *in situ* hybridizations performed on both *D. guanche* (GI_16) and *D. subobscura* (*ch cu*) polytene chromosomes. Hybridization signals at the inversion breakpoints are marked with red arrows. Their cytological location is indicated according to the Kunze-Mühl and Müller (1958) map of *D. subobscura*. C, centromere; T, telomere.

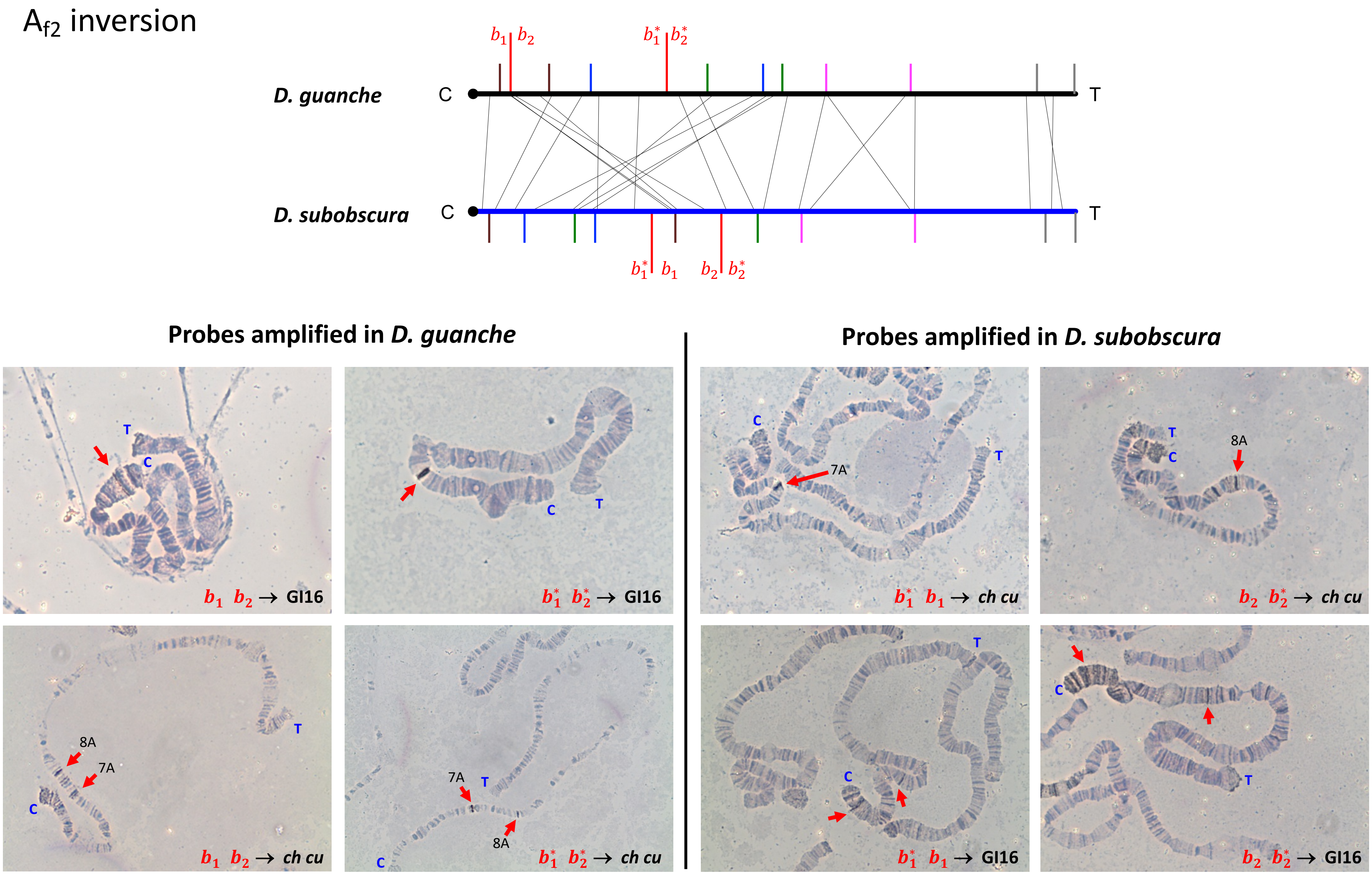

Supplementary Figure S5. ***In situ* hybridization results of the A_{f2} inversion breakpoints probes in *D. guanche* and *D. subobscura*.** Upper part, schematic representation of the A_{f2} inversion using the same notation for breakpoint regions than in Figure 1. Lower part, results of the *in situ* hybridizations performed on both *D. guanche* (GI_16) and *D. subobscura* (*ch cu*) polytene chromosomes. Hybridization signals at the inversion breakpoints are marked with red arrows. Their cytological location is indicated according to the Kunze-Mühl and Müller (1958) map of *D. subobscura*. C, centromere; T, telomere.

A_{f3} inversion

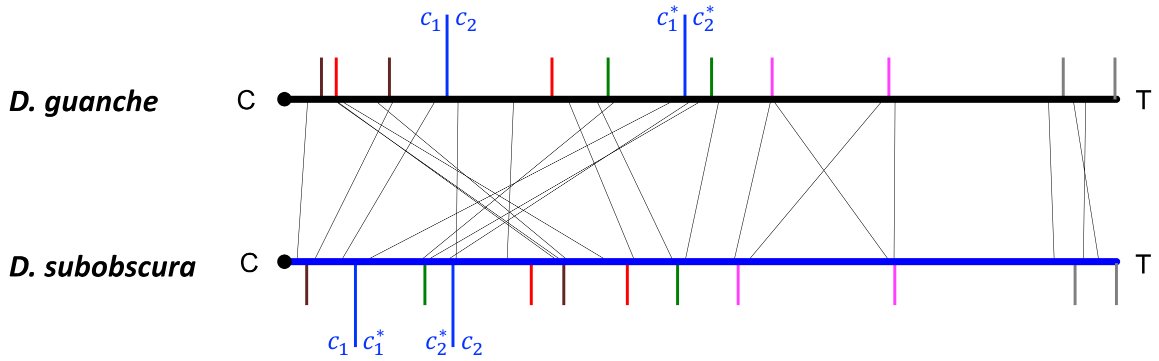

Probes amplified in *D. guanche*

Probes amplified in *D. subobscura*

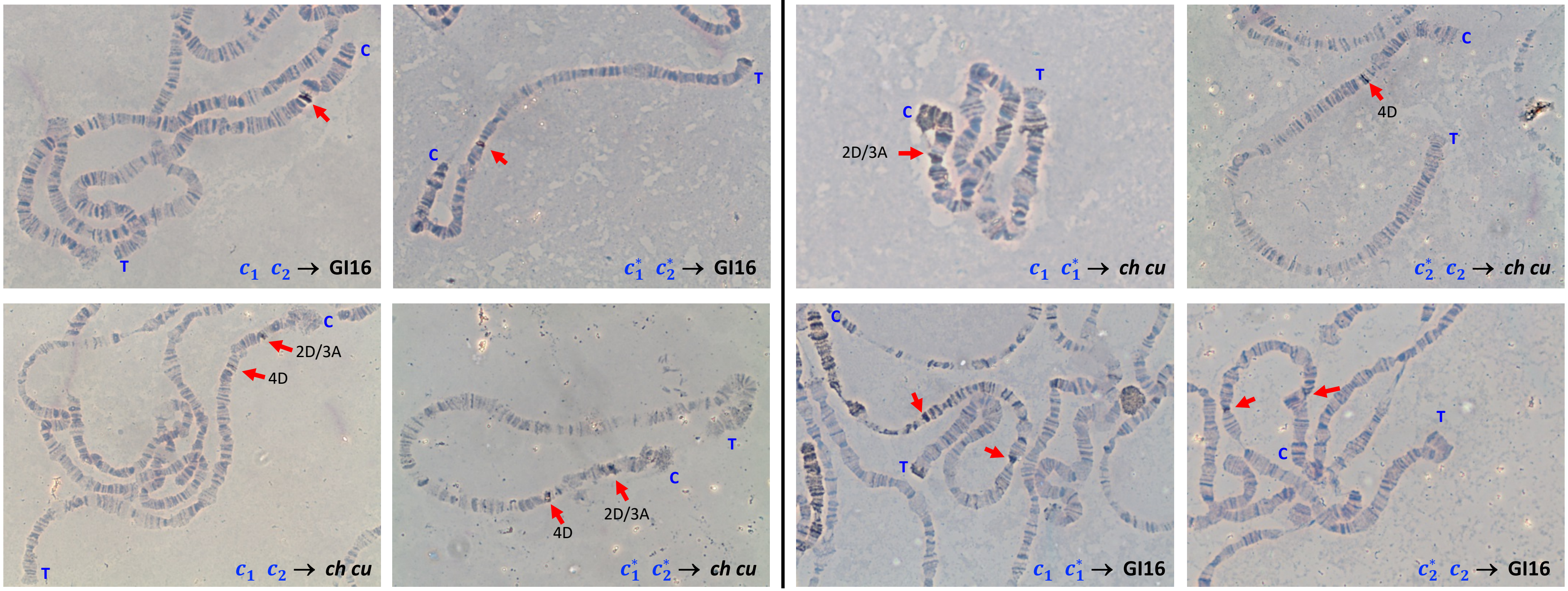

Supplementary Figure S6. *In situ* hybridization results of the A_{f3} inversion breakpoints probes in *D. guanche* and *D. subobscura*. Upper part, schematic representation of the A_{f3} inversion using the same notation for breakpoint regions than in Figure 1. Lower part, results of the *in situ* hybridizations performed on both *D. guanche* (GI_16) and *D. subobscura* (*ch cu*) polytene chromosomes. Hybridization signals at the inversion breakpoints are marked with red arrows. Their cytological location is indicated according to the Kunze-Mühl and Müller (1958) map of *D. subobscura*. C, centromere; T, telomere.

A_{f4} inversion

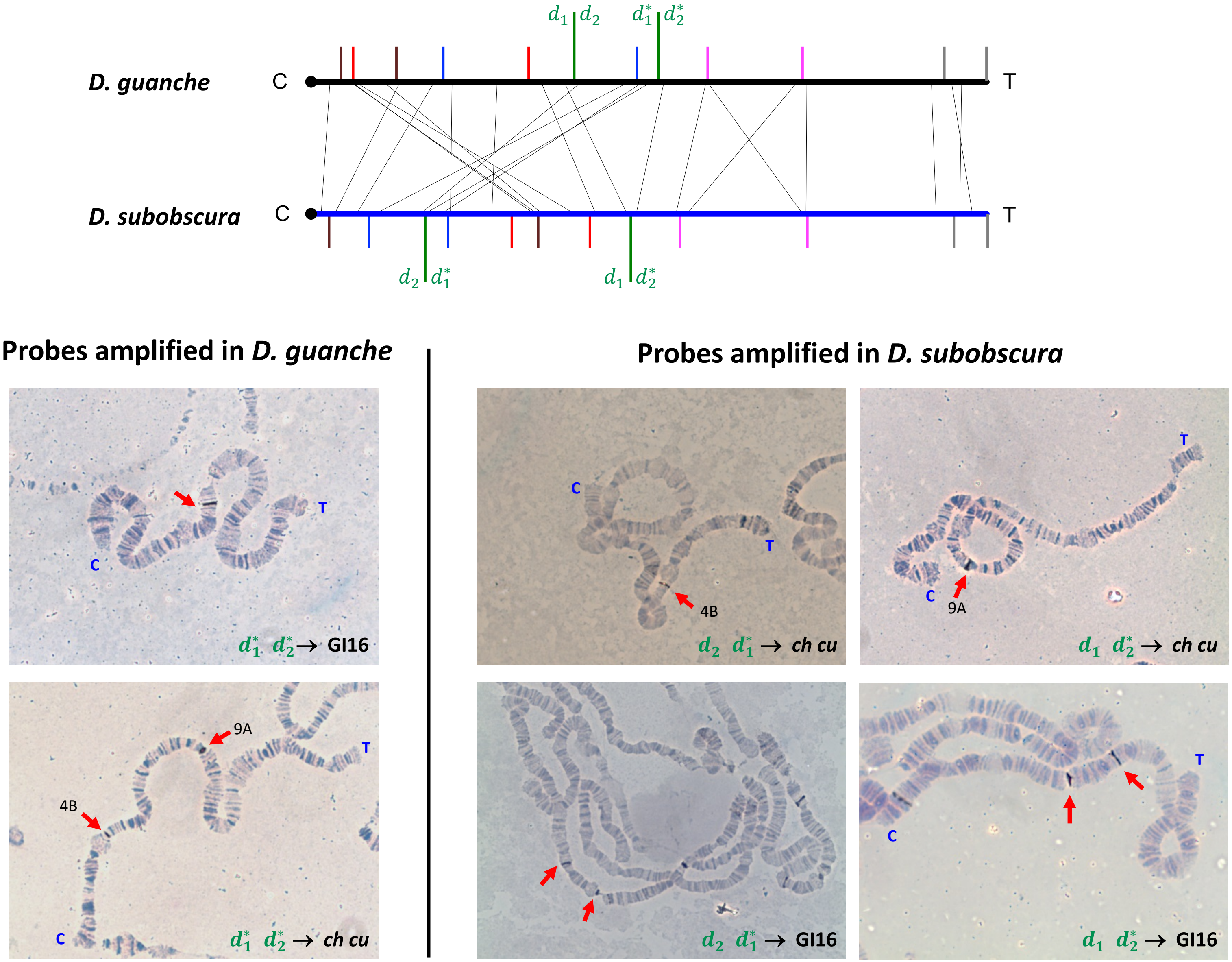

Supplementary Figure S7. *In situ* hybridization results of the A_{f4} inversion breakpoints probes in *D. guanche* and *D. subobscura*. Upper part, schematic representation of the A_{f4} inversion using the same notation for breakpoint regions than in Figure 1. Lower part, results of the *in situ* hybridizations performed on both *D. guanche* (GI_16) and *D. subobscura* (*ch cu*) polytene chromosomes. Hybridization signals at the inversion breakpoints are marked with red arrows. Their cytological location is indicated according to the Kunze-Mühl and Müller (1958) map of *D. subobscura*. C, centromere; T, telomere.

A_{f5} inversion

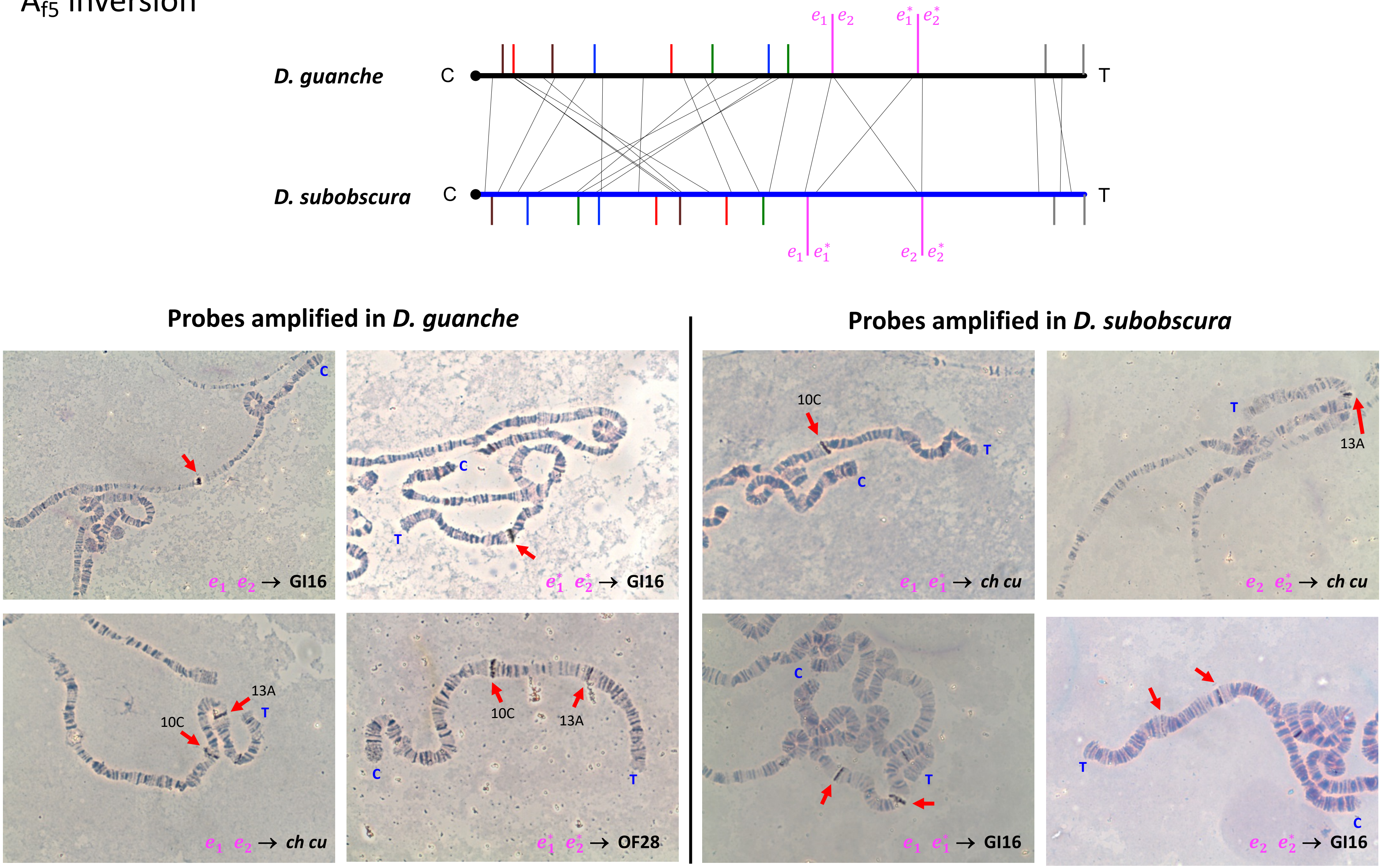

Supplementary Figure S8. *In situ* hybridization results of the A_{f5} inversion breakpoints probes in *D. guanche* and *D. subobscura*. Upper part, schematic representation of the A_{f5} inversion using the same notation for breakpoint regions than in Figure 1. Lower part, results of the *in situ* hybridizations performed on both *D. guanche* (GI_16) and *D. subobscura* (*ch cu* or OF28) polytene chromosomes. Hybridization signals at the inversion breakpoints are marked with red arrows. Their cytological location is indicated according to the Kunze-Mühl and Müller (1958) map of *D. subobscura*. C, centromere; T, telomere.
